# Prediction of slope failure in open-pit mines using a novel hybrid artificial intelligence model based on decision tree and evolution algorithm

**DOI:** 10.1038/s41598-020-66904-y

**Published:** 2020-06-18

**Authors:** Xuan-Nam Bui, Hoang Nguyen, Yosoon Choi, Trung Nguyen-Thoi, Jian Zhou, Jie Dou

**Affiliations:** 1 Department of Surface Mining, Mining Faculty, Hanoi University of Mining and Geology, Duc Thang, Bac Tu Liem, Hanoi Vietnam; 2 Center for Mining, Electro-Mechanical Research, Hanoi University of Mining and Geology, Duc Thang, Bac Tu Liem, Hanoi Vietnam; 3 Institute of Research and Development, Duy Tan University, Da Nang, 550000 Vietnam; 4 Department of Energy Resources Engineering, Pukyong National University, Busan, 48513 Korea; 5 Division of Computational Mathematics and Engineering, Institute for Computational Science, Ton Duc Thang University, Ho Chi Minh City, Vietnam; 6 Faculty of Civil Engineering, Ton Duc Thang University, Ho Chi Minh City, Vietnam; 7 School of Resources and Safety Engineering, Central South University, Changsha, Hunan 410083 China; 8 Civil and Environmental Engineering, Nagaoka University of Technology, 1603-1, Kami-Tomioka, Nagaoka, Niigata, 940-2188 Japan

**Keywords:** Natural hazards, Engineering

## Abstract

In this study, the objective was to develop a new and highly-accurate artificial intelligence model for slope failure prediction in open-pit mines. For this purpose, the M5Rules algorithm was combined with a genetic algorithm (GA) in a novel hybrid technique, named M5Rules–GA model, for slope stability estimation and analysis and 450-slope observations in an open-pit mine in Vietnam were modeled using the Geo-Studio software based on essential parameters. The factor of safety was used as the model outcome. Artificial neural networks (ANN), support vector regression (SVR), and previously introduced models (such as FFA-SVR, ANN-PSO, ANN-ICA, ANN-GA, and ANN-ABC) were also developed for evaluating the proposed M5Rules–GA model. The evaluation of the model performance involved applying and computing the determination coefficient, variance account for, and root mean square error, as well as a general ranking and color scale. The results confirmed that the proposed M5Rules–GA model is a robust tool for analyzing slope stability. The other investigated models yielded less robust performance under the evaluation metrics.

## Introduction

**Figure 1:**
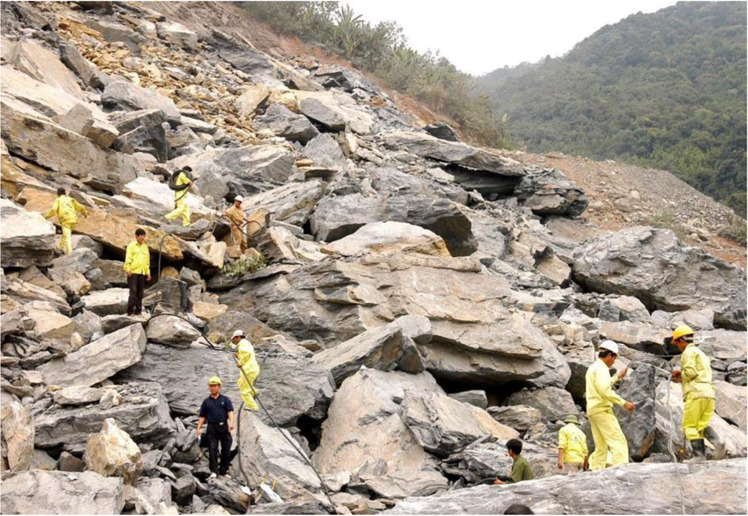
Slope collapse disaster in an open-pit mine in Vietnam.

Slope collapse is a critical hazard in open-pit mines as it can be of any scale, small or large, and directly affect people, equipment, and production processes (Fig. 1). Therefore, analysis and calculation of slope stability are of vital importance for preventing disasters that occur because of such instabilities.

Numerical methods^1,2^ and three-dimensional techniques^3^ for slope stability analysis were applied to various geological structures^,^. Wei, *et al*.^4^ combined the generalized Hoek-Brown and strength reduction method to evaluate the stability of slopes in rock mass. Many types of seismic actions were also investigated and assessed for the stability of slopes in different conditions^5–9^. However, owing to the complexity of geological structures, slope stability is a challenging aspect for large open-pit mining projects^10^.

Furthermore, as soil layers exhibit heterogeneous characteristics, geotechnical and geological uncertainties can worsen the poor estimation of slope stability^11,12^. The random finite element method (RFEM) and limit equilibrium method (LEM), as well as other modeling methods based on finite elements and stochastic simulation, were typically applied to calculate slope stability^13–16^.

In recent years, more advanced computational techniques have been widely applied in many fields^17–20^, particularly in the prediction of landslides including slope stability^21–23^. Artificial intelligence (AI) is a powerful tool capable of replacing traditional methods such as slope stability and landslide^24–26^, blast-induced problems (e.g., ground vibration, air over-pressure, fly-rock, rock fragmentation, etc.)^27–32^, optimization in mine planning^33,34^, and geology and geophysics^35–37^. For slope stability prediction, Qi and Tang^38^ developed six different soft computing models based on a meta-heuristic algorithm (i.e., firefly optimization) and machine learning algorithms (i.e., random forest, logistic regression, gradient boosting machine, support vector machine, decision tree, and multilayer perceptron neural network). A promising result was found in their study for predicting slope stability when the area under the receiver operating characteristic curve reached up to 0.967. Sakellariou and Ferentinou^39^ introduced an artificial neural network (ANN) model that used geometrical and geotechnical parameters to predict the factor of safety (FOS) based on their database of experiments. In another study, Samui^40^ applied support-vector machine (SVM) for slope stability analysis using a database of practical investigations. Choobbasti, *et al*.^41^ also conducted similar work with ANN models. Despite the high appreciation from researchers for the effectiveness of AI techniques in predicting slope stability, experimental data are often insufficient because of time and cost constraints.

To overcome the abovementioned limitations, various simulation software based on RFEM and LEM methods, among others, were introduced (such as OptumG2 and Geo-Studio)^42,43^. Based on these tools, slope stability prediction was analyzed and accurately evaluated for many models. Moreover, scientists have applied a big-data approach to AI for slope stability analysis^44–46^. Chakraborty and Goswami^47^ simulated 200 cases with different shear strengths and geometric parameters to evaluate slope stability using ANN and multiple linear regression (MLR). Jellali and Frikha^48^ used OptumG2 to generate 30,000 elements and predicted slope stability using the particle swarm optimization (PSO) algorithm with promising results. Mojtahedi, *et al*.^49^ applied the Monte Carlo technique using Geo-Studio software with 224 datasets. Saleh^50^ applied ANN to a database of 2,180 simulated slope cases using Geo-Studio software. Qi and Tang^38^ attempted to develop six soft computing models using optimization approaches based on firefly algorithms (FFA), concluding that an FFA–SVM model was the best. Koopialipoor, *et al*.^51^ applied various hybrid AI models, such as ANN-PSO, ANN-ICA (imperialist competitive algorithm), ANN-GA (genetic algorithm), and ANN-ABC (artificial bee colony), based on OptumG2 software analysis results. They found that the ANN-PSO model provided better performance than the other models. Gao, *et al*.^52^ successfully developed a promising hybrid model called ICA–ANN based on the combination of ANN and ICA with 400 OptumG2 simulations. Qian, *et al*.^53^ also performed similar work for forecasting slope stability based on OptumG2 software.

The review of previous works reveals that AI techniques are widely applied in slope stability prediction and analysis. However, such methods are not applied in all areas/regions. Furthermore, many AI models and techniques are yet to be investigated. To promote continued improvements in safety, development of knowledge, and enhancing slope stability, prediction performance in other areas is necessary. Therefore, a novel hybrid model, namely M5Rules–GA, for predicting slope stability (i.e., FOS) using a genetic algorithm (GA) and M5Rules was proposed and investigated in this study. It is worth mentioning that the team of authors developed the M5Rules-GA model for predicting the energy efficiency of buildings (i.e., heating load) with high accuracy^54^. However, it is not taking into account to predict and evaluate the stability of slopes. Furthermore, the performance, as well as the parameters of the M5Rules-GA model, are different based on different databases. Therefore, the M5Rules-GA model was investigated to predict slope stability herein and it is considered as a novel model in this field. Besides, several ANNs, support-vector regression (SVR), and previously introduced slope stability prediction models (such as FFA-SVR, ANN-PSO, ANN-ICA, ANN-GA, and ANN-ABC) were also implemented for a comprehensive comparison of the proposed M5Rules–GA model. There were 450 simulations of open-pit mine slopes in Vietnam, as a database for predicting slope stability.

## Background: M5Rules and GA

In this study, eight AI techniques were used to develop slope failure predictive models, including ANN, SVR, M5Rules, PSO, FFA, ICA, ABC, and GA. However, the details for ANN, PSO, FFA, ICA, ABC, and SVR techniques were presented in many previous works^19,55–60^. Therefore, these details are not included in the present study. This section highlights the background of M5Rules and GA for developing the new hybrid M5Rules–GA model.

### M5Rules

**Figure 2:**
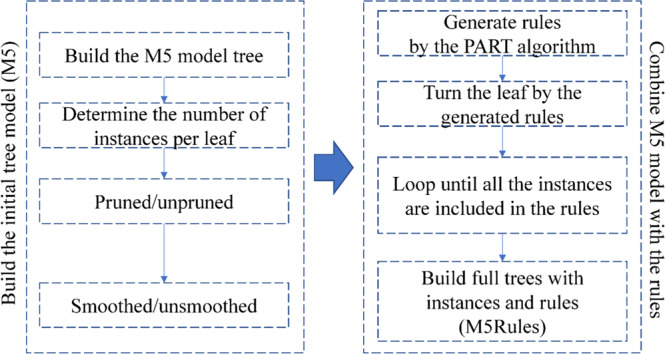
Workflow of the M5Rules model.

M5Rules is well-known as an enhanced model of the M5 model with rules^61^. It is a type of decision tree algorithm in machine learning that can be applied for regression and classification problems^62,63^. In regression, M5Rules is based on a combination of regression tree models^64^. In addition, the partial and regression tree (PART) algorithm is applied to generate the rules for the M5 tree model^65^. These rules can improve the performance of the M5 tree model rather than the regression tree models, and it is called M5Rules. In M5Rules, the tree can be developed based on the four following options: pruned/unpruned tree; smoothed/unsmoothed predictions; build regression tree/rule; and define the minimum number of instances per leaf^66^. The workflow for the development of the M5Rules model is shown in Fig. 2.

### Genetic algorithm

**Figure 3:**
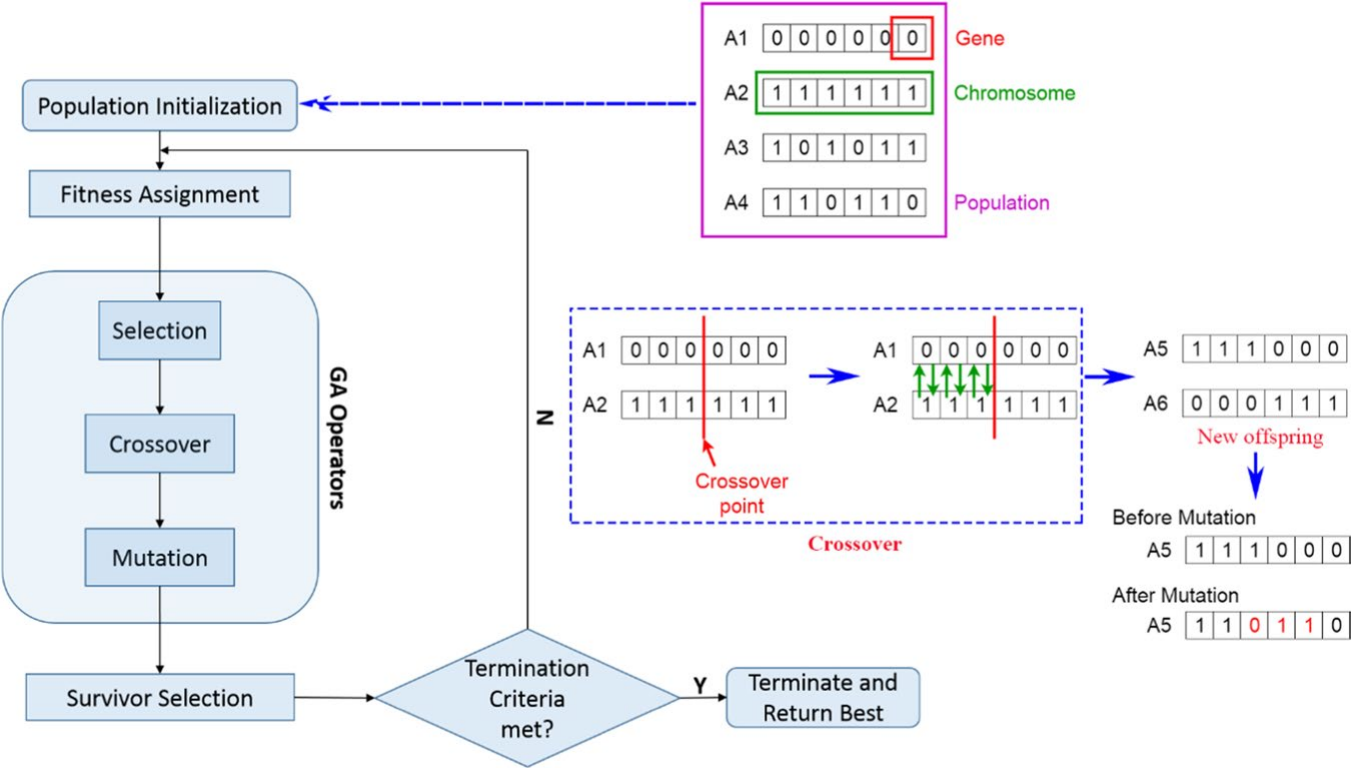
Description of GA with operators.

Meta-heuristic algorithms are well-known as robust algorithms for optimization problems. Among those, the GA has one of the dominant performances in optimization based on the theory of Darwin^67,68^. Four steps are conducted in GA for finding an optimal function: genetics, mutation, natural selection, and crossover. Before implementing an optimization of an objective function, GA needs an initial population and their fitness are calculated^69,70^. Note that the number of population of individuals is generated heuristically or randomly^71^. In GA, the quality of the population can be improved by the selection operator. Subsequently, two individuals are generated with higher fitness using the crossover operator. Mutation operators can create a new generation with better performance in the population by randomly modifying some genes^72^. It is worth mentioning that replacement strategies can be applied to replace the current generation by newly generated offsprings. There are two main types of replacement: generational and steady-state replacements^73^. In addition, other related replacement strategies, such as elitism, delete n-last, delete n, random replacement, weak parent replacement, and both parents replacement, can be applied for replacement of generation in GA^74^. The structure and the framework of the GA are simulated in Fig. 3.

In GA, the cycle of operators (i.e., selection, crossover, and mutation) is employed and looped. To end the algorithm, two stopping conditions can be applied as follows:

1. Tcn the structure of the chromosome.
2. The change in fitness from newly generated offsprings is less than a specified constant.

### Framework of M5Rules–GA model for slope stability analysis

**Figure 4:**
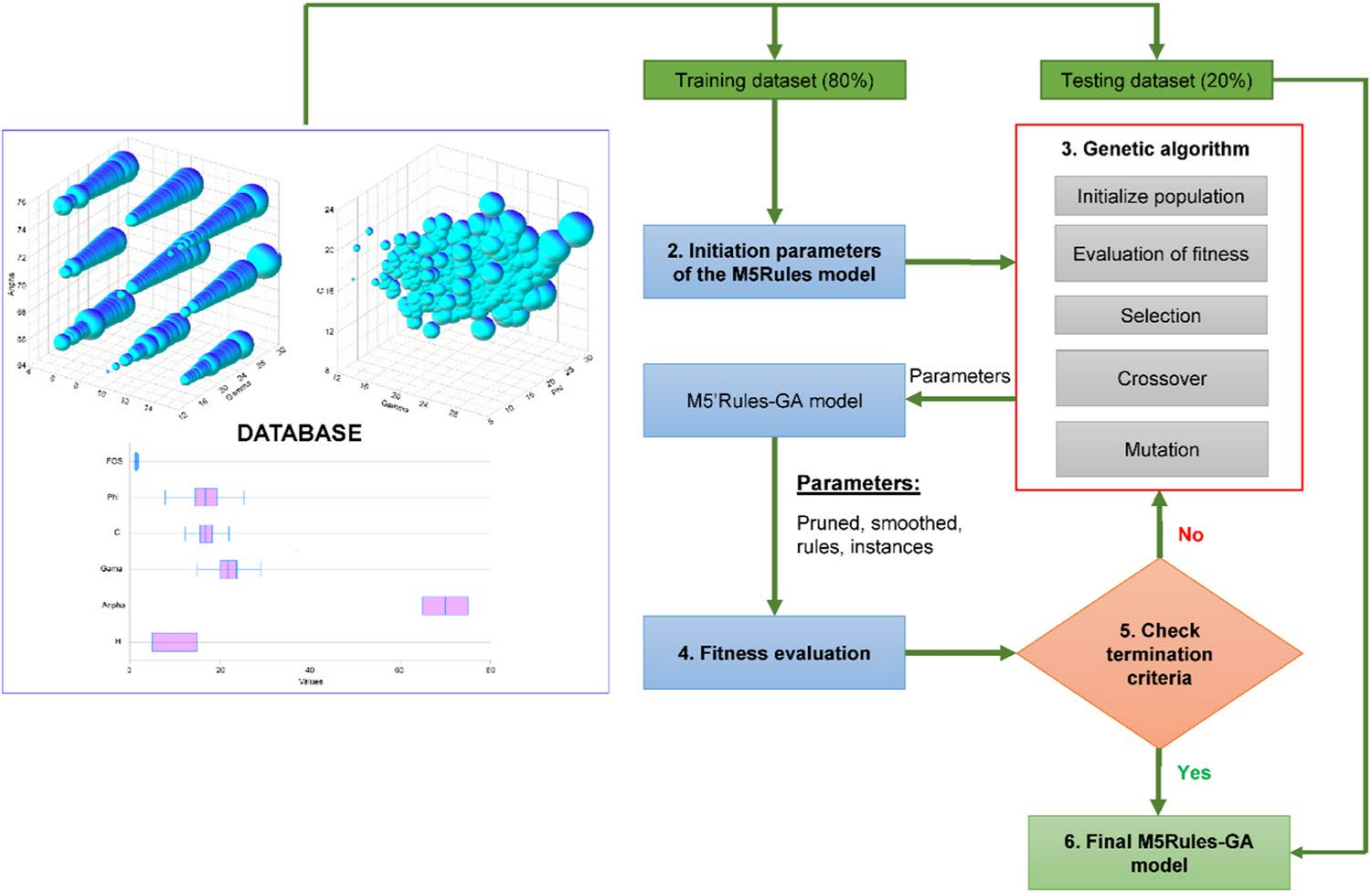
Flow chart of M5Rules–GA model for analyzing slope stability.

In this section, the M5Rules–GA model, which is the slope stability prediction model proposed in this study, is presented and highlighted. The performance of the M5Rules model is determined by its parameters. Pruning and smoothing tasks can be applied during the development of the M5 tree model. Additionally, rules and the number of instances per leaf are important parameters affecting the performance of the M5Rules model. Therefore, the GA is applied to optimize the parameters of the M5Rules model: *pruned, smoothed*, *rules*, and *the number of instances per leaf*. Note that the PART algorithm generates rules for the M5 tree model. With each round, GA searches the M5Rules model parameter values with the M5Rules model performance evaluated using a fitness function (i.e., root mean square error (RMSE)). The search process is performed until the optimal M5Rules model is found, i.e., the model with the most extreme fitness function value. The framework of the M5Rules–GA model is illustrated in Fig. 4.

### Statistical criteria

To evaluate the accuracy and error of the developed models, RMSE, determination coefficient (R^2^), variance account for (VAF), and color intensity were applied based on the measured and predicted values on both training and testing phases. They were calculated according to Eqs. (1–3).

1 $$\text{RMSE}=\sqrt{\frac{1}{n}\mathop{\sum }\limits_{i=1}^{n}{({y}_{i}-{\hat{y}}_{i})}^{2}}$$

2 $${\text{R}}^{\text{2}}=1-\frac{\sum _{i}({y}_{i}-{\hat{y}}_{i})}{\sum _{i}({y}_{i}-\bar{y})}$$

3 $$\text{VAF}=\left(1-\frac{\mathrm{var}({y}_{i}-{\hat{y}}_{i})}{\mathrm{var}({y}_{i})}\right)\times 100$$

where *n* represents the number of instances, and $\overline{y}$, ${y}_{i}$, and ${\hat{y}}_{i}$ represent the average, measured, and modeled values of the response variable, respectively.

## Case study

**Figure 5:**
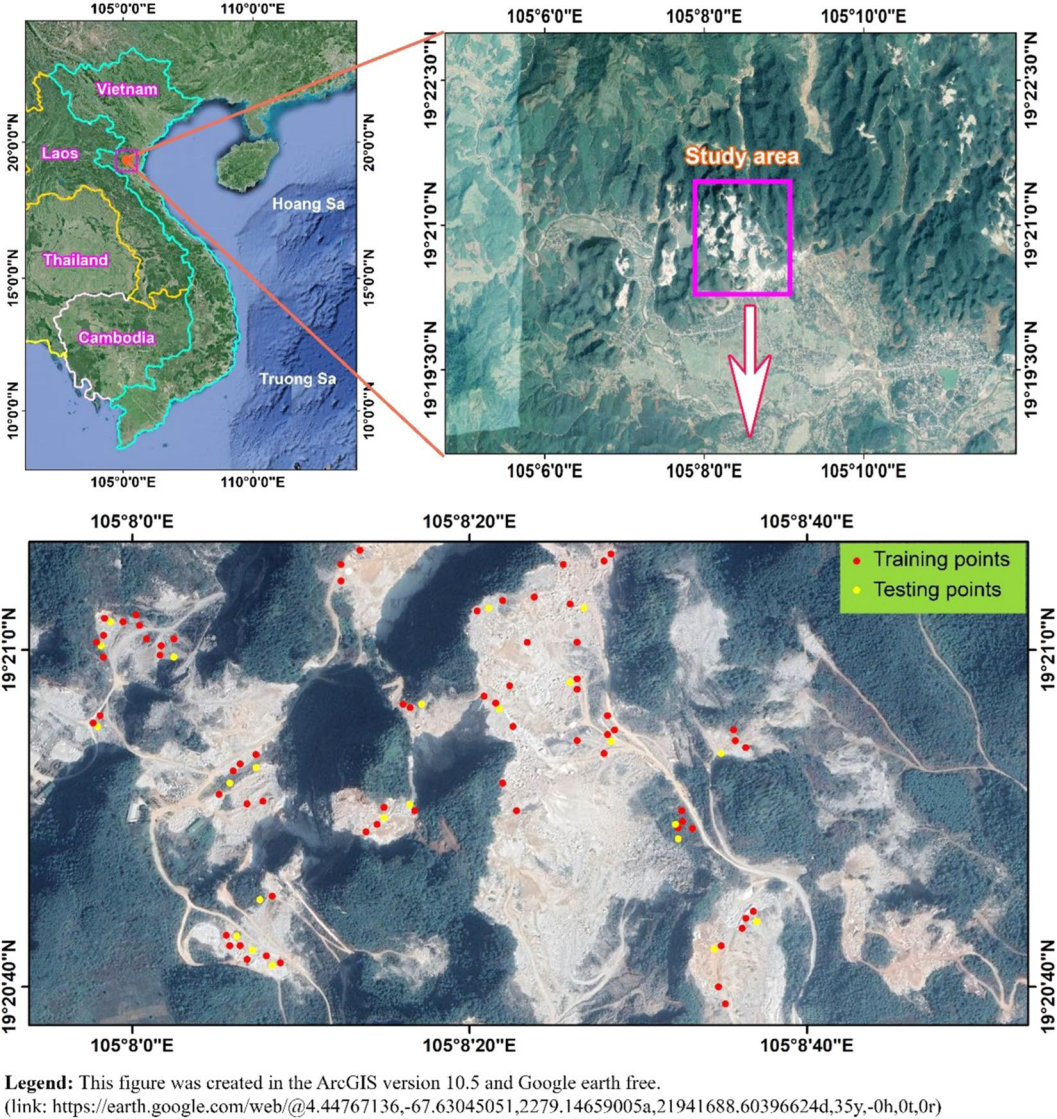
Study area and its landscape via Google Earth.

For assessing the performance of the proposed M5Rules–GA model in practical engineering, a quarry mine in Vietnam was selected as a case study (Fig. 5). The parameters used to predict the stability of the slope included bench height (H), unit weight ($\gamma$), cohesion (*C*), angle of internal friction (*φ*), and slope angles ($\alpha$); the FOS was assigned as the output parameter. Properties of the dataset used are detailed in Table 1.

**Table 1:** Summary of the features of inputs and output.

Features	H	α	γ	C	φ	FOS
Min.	5	65	12.83	11.22	7.3	0.76
1st Qu.	5	65	20.09	15.6	14.5	1.29
Median	10	70	21.85	16.85	16.8	1.41
Mean	10	70	21.87	16.96	16.8	1.42
3rd Qu.	15	75	23.71	18.3	19.3	1.54
Max.	15	75	31.53	23.34	27.8	1.98

**Figure 6:**
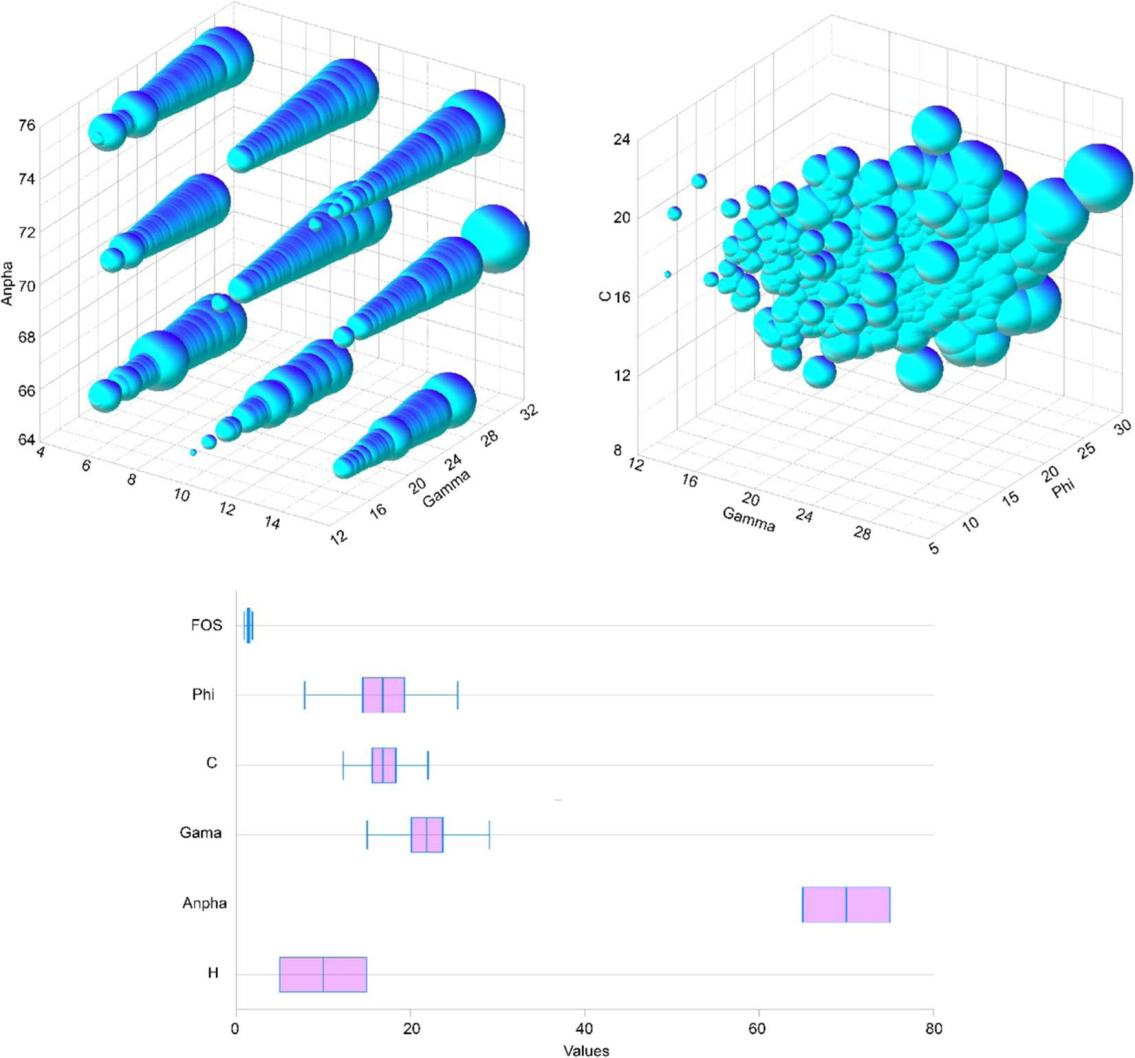
Visualization of slope stability database.

As recommended by previous researchers, $\gamma$, $\alpha$, H,, and *C* are the most influential parameters that have impacts on the FOS^58,75^. Therefore, these factors were provided to the Geo-Studio (version 2019) for the computations of FOS values. According to Zhou, *et al*.^76^, the slope is stable at FOS > 1. However, according to Sakellariou and Ferentinou^39^, the slope is stable only at FOS $\ge$ 1.2. Thus, for safety in mining, FOS was assigned at least 1.2. Therefore, the slopes will be stable if $FOS\ge 1.2$, and fail if $FOS < 1.2$. In this study, 450 simulations were conducted in the laboratory using Geo-Studio software based on the working conditions. The simulation results showed that the slopes of the study site included both stability and failure, i.e., $0.76\le FOS\le 1.98$. The datasets used in this study are visualized in Fig. 6, and summarized in Table 1.

## Results and discussions

To develop the models, the FOS database needs to be prepared and normalized. Accordingly, the dataset used should be split into two phases. As recommended in previous studies^56,77,78^, 80% of the database was used for the training of the models; the remaining 20% was used to assess the models’ performance. The training dataset was randomly selected, and all the abovementioned models were developed based on the same training phase and tested using the same testing phase.

**Figure 7:**
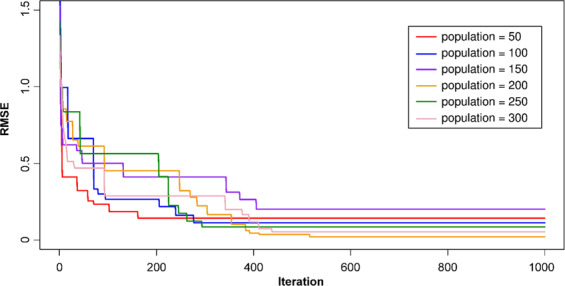
Optimizing the M5Rules model with the GA procedure.

For the development of the M5Rules–GA model, the step-by-step approach shown in Fig. 4 was applied. An initialization of the M5Rules model was developed based on its parameters and the training dataset, as the first step. To improve the model’s performance, the 10-fold cross-validation resampling technique was used. Next, the GA’s parameters were established as the second step: mutation probability (*Pm*), crossover probability (*Pc*), number variable (*n*), and number of populations (*p*). *Pm*, *Pc*, and *n* were set to 0.1, 0.8, and 5, respectively, and the values for *p* were set to 50, 100, 150, 200, 250, and 300. Additionally, the steady-state replacement method^79^ was applied in GA. RMSE was used as the fitness function, according to Eq. 1. The maximum number of iterations was set to 1000 to ensure the finding of the best values of the M5Rules model with the lowest RMSE value (i.e., best fitness value). Figure 7 shows that the M5Rules–GA model reached the best performance with *p* = 200 at the iteration of 412 (RMSE = 0.0218).

For SVR modeling, a kernel function was applied (i.e., radial basis function) with *σ* and *C* used as the main parameters for controlling SVR model performance. A 10-fold cross-validation^80^ was applied to increase the accuracy while preventing overfitting or underfitting of the SVR model. Furthermore, the Box-Cox transformation technique^81^ was applied to reduce the skewness of the data. A trial and error approach with various SVR models was conducted to determine the best SVR model for this study. The best SVR model for analyzing slope stability was found at $\sigma$ = 0.014 and *C* = 276.385.

**Figure 8:**
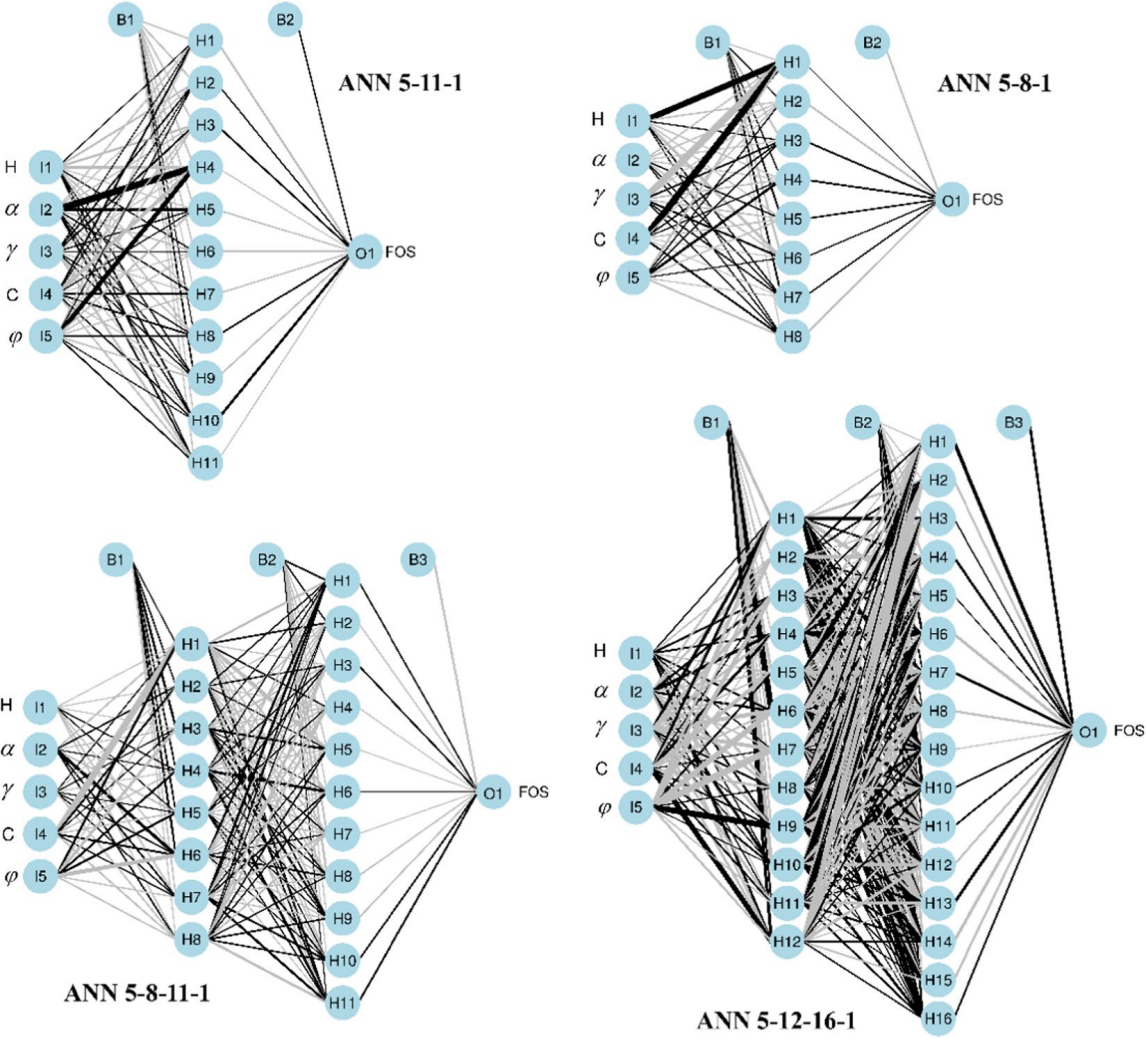
ANN models for analyzing slope stability in this study.

For ANN models, hidden model layers resist definition or explicit explanation. However, according to previous works^28,82–84^, ANNs with one or two hidden layer(s) can solve most problems. Therefore, a trial and error approach was conducted to find the best ANN models with one or two hidden layer(s). The min-max scale technique (i.e., [0,1]) was used as a normalization method for the dataset to avoid overfitting of the ANN models. Eventually, four ANN models were established: ANN 5-8-1, ANN 5-11-1, ANN 5-8-11-1, and ANN 5-12-16-1, called ANN 1, ANN 2, ANN 3, and ANN 4, respectively. Their structures can be seen in Fig. 8. In addition, the FFA-SVR, ANN-PSO, ANN-ICA, ANN-GA, and ANN-ABC models introduced by previous researchers were taken into consideration to predict FOS and compare with the developed M5Rules-GA model. To predict the stability of the slope, the FFA-SVR, ANN-PSO, ANN-ICA, ANN-GA, and ANN-ABC models were also developed through two phases: (1) Initializing an ANN model with initial weights and biases; (2) Optimizing the initialization ANN model by the FFA algorithm. Indeed, the weights and biases of the established ANN model were optimized by the FFA algorithm aiming to improve the accuracy of the initialization ANN model. In other words, the role of the pairs ANN and M5Rules, FFA, ABC, PSO, ICA and GA are the same in this study. It should be noted that the Box-Cox transformation technique was applied to preprocess the dataset aiming to prevent overfitting of the M5Rules and SVR models. For the ANN models, the MinMax [0,1] technique was used to normalized the dataset for the same purposes as those of M5Rules and SVR models. Ultimately, the performance of the slope stability evaluation models on both training and testing datasets are computed in Table 2.

**Table 2:** Performance of the slope stability evaluation models.

Model	Training		Testing	
	RMSE	R^2^	RMSE	R^2^
M5Rules-GA	0.022	0.985	0.024	0.983
ANN 5-8-1	0.033	0.971	0.031	0.970
ANN 5-11-1	0.033	0.973	0.030	0.975
ANN 5-8-11-1	0.032	0.971	0.027	0.978
ANN 5-12-16-1	0.033	0.970	0.032	0.969
SVR	0.032	0.975	0.030	0.974
FFA-SVR	0.027	0.978	0.029	0.976
ANN-PSO	0.026	0.980	0.026	0.980
ANN-ICA	0.028	0.977	0.029	0.976
ANN-GA	0.029	0.980	0.027	0.979
ANN-ABC	0.030	0.980	0.028	0.978

Based on Table 2, it is worth mentioning that all the models performed very well in predicting the slope stability without overfitting. However, it is hard to recognize which model is the best among them. Therefore, once the models were well-established based on the training dataset, their performance should be tested using the testing phase with performance indices (i.e., RMSE, R^2^, VAF, a general ranking, and color range). The purpose of using multiple metrics, ranking, and color intensity methods is to recognize the best model in those of the developed models. Furthermore, the testing dataset is taken into account as the new dataset in practical; thus, evaluating the performance of the models on the testing dataset will provide an overview of the reliability of the models in practice. Also, to evaluate the performance of the models through the training time (runtime), the total time of training of the models was calculated in Table 3. It is worth mentioning that the runtime of the models highly depends on the hardware of the computer used. Herein, a workstation computer with the Intel(R) Xeon(R) dual CPU X5675 3.07 GHz, 24 GB RAM, and K5000 VGA (5.0 GB) was used to train the models. The testing results of the predictive models, as well as their ranking, are listed in Table 3.

**Table 3:** Testing the performance of slope stability predictive models.

Model	Performance of the models				Ranking			
	Runtime	RMSE	R^2^	VAF	Rank for RMSE	Rank for R^2^	Rank for VAF	Total ranking
M5Rules-GA	157.201	0.024	0.983	98.260	11	11	11	33
ANN 5-8-1	31.225	0.031	0.970	97.037	2	2	2	6
ANN 5-11-1	32.033	0.030	0.975	97.280	3	4	3	10
ANN 5-8-11-1	62.779	0.027	0.978	97.707	8	7	8	23
ANN 5-12-16-1	94.542	0.032	0.969	96.840	1	1	1	3
SVR	125.332	0.030	0.974	97.320	3	3	4	10
FFA-SVR	188.382	0.029	0.976	97.517	5	5	5	15
ANN-PSO	217.677	0.026	0.980	97.907	10	10	10	30
ANN-ICA	250.701	0.029	0.976	97.552	5	5	6	16
ANN-GA	282.772	0.027	0.979	97.751	8	9	9	26
ANN-ABC	314.388	0.028	0.978	97.647	7	7	7	21

Based on Table 3, it is clear that the training time of the hybrid models is higher than the single models. This problem is due to the calculation volume of the hybrid models is higher than the individual models with many repetitions. Of those, the training time of the M5Rules-GA model is lowest among the hybrid modes developed (i.e., M5Rules-GA, FFA-SVR, ANN-PSO, ANN-ICA, ANN-GA, ANN-ABC) with 157.201 seconds. Whereas, the training time of the ANN-PSO model is higher than those of the M5Rules-GA models even though its accuracy is slightly lower than the M5Rules-GA model. Another hybrid model based on the GA, i.e., ANN-GA, also taken more training time than the M5Rules-GA model (i.e., 282.772 seconds). They showed that the optimization of M5Rules is faster than the ANN model.

**Figure 9:**
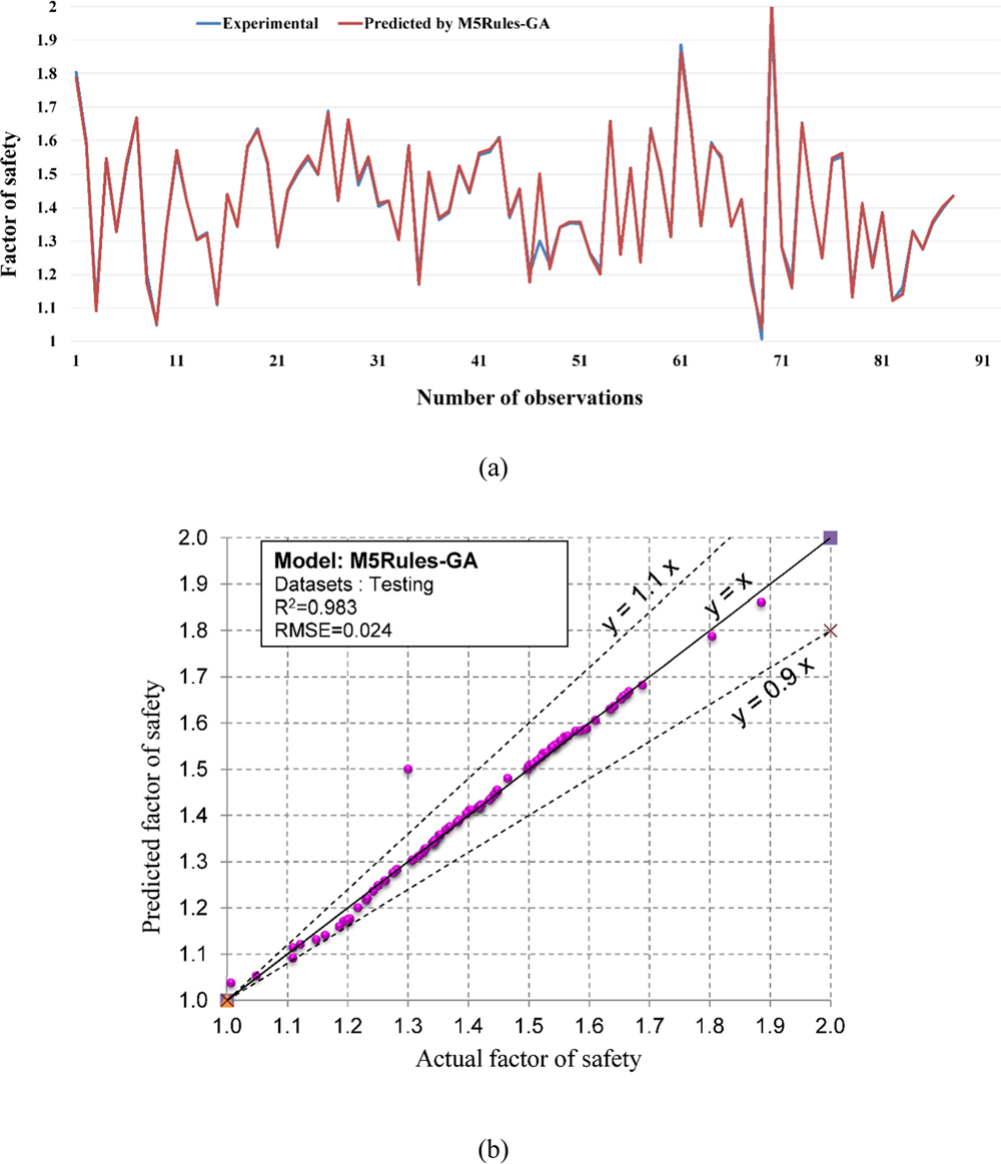
The accuracy and the converging of the M5Rules-GA model in predicting FOS. (**a**) Different between the actual and predicted FOS values. (**b**) Correlation analyses of the actual and predicted FOS values.

**Figure 10:**
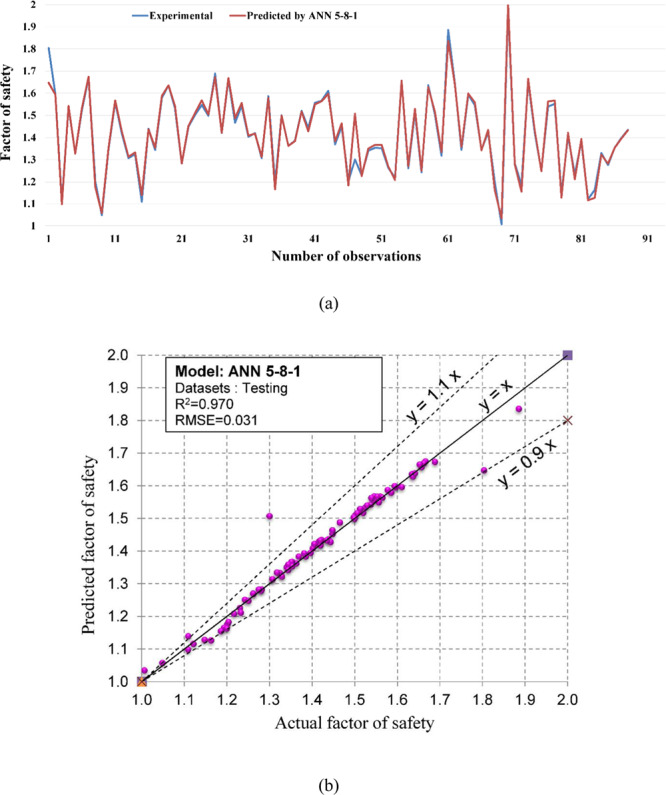
The accuracy and the converging of the ANN 5-8-1 model in predicting FOS. (**a**) Different between the actual and predicted FOS values. (**b**) Correlation analyses of the actual and predicted FOS values.

**Figure 11:**
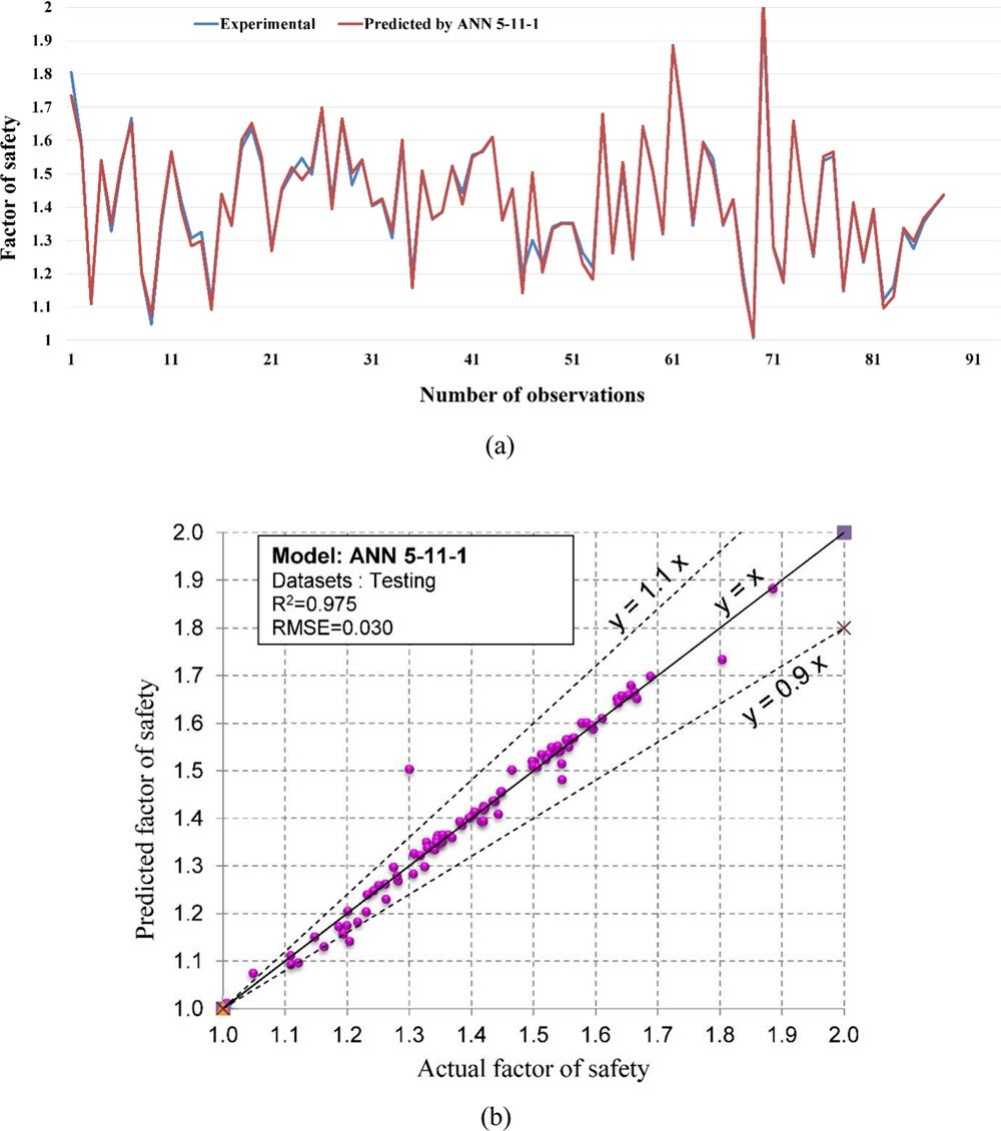
The accuracy and the converging of the ANN 5-11-1 model in predicting FOS. (**a**) Different between the actual and predicted FOS values. (**b**) Correlation analyses of the actual and predicted FOS values.

**Figure 12:**
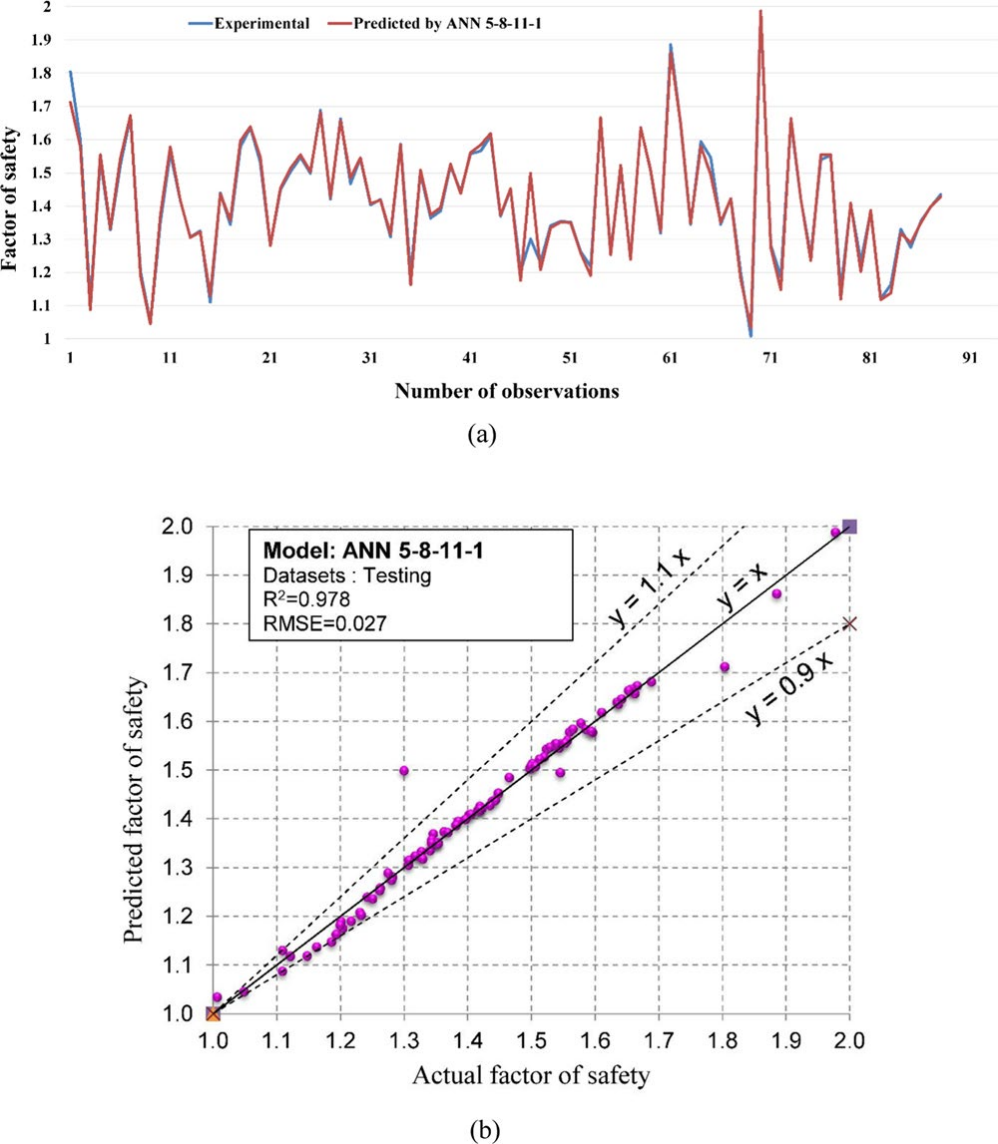
The accuracy and the converging of the ANN 5-8-11-1 model in predicting FOS. (**a**) Different between the actual and predicted FOS values. (**b**) Correlation analyses of the actual and predicted FOS values.

**Figure 13:**
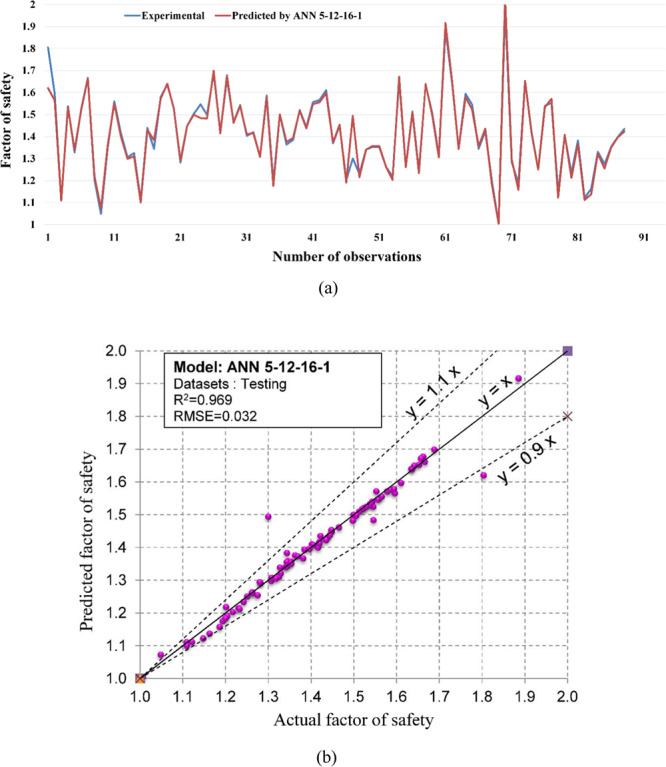
The accuracy and the converging of the 5-12-16-1 model in predicting FOS. (**a**) Different between the actual and predicted FOS values. (**b**) Correlation analyses of the actual and predicted FOS values.

**Figure 14:**
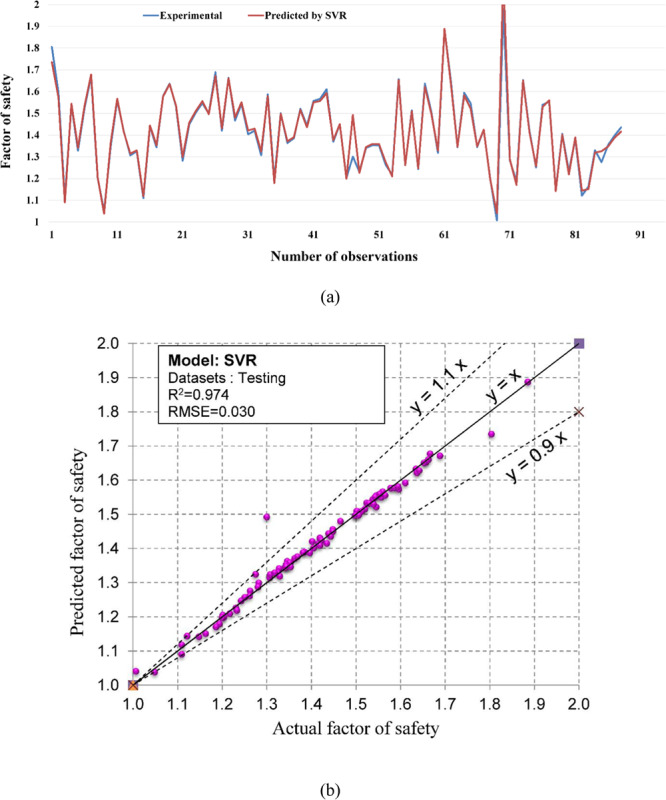
The accuracy and the converging of the SVR model in predicting FOS. (**a**) Different between the actual and predicted FOS values. (**b**) Correlation analyses of the actual and predicted FOS values.

**Figure 15:**
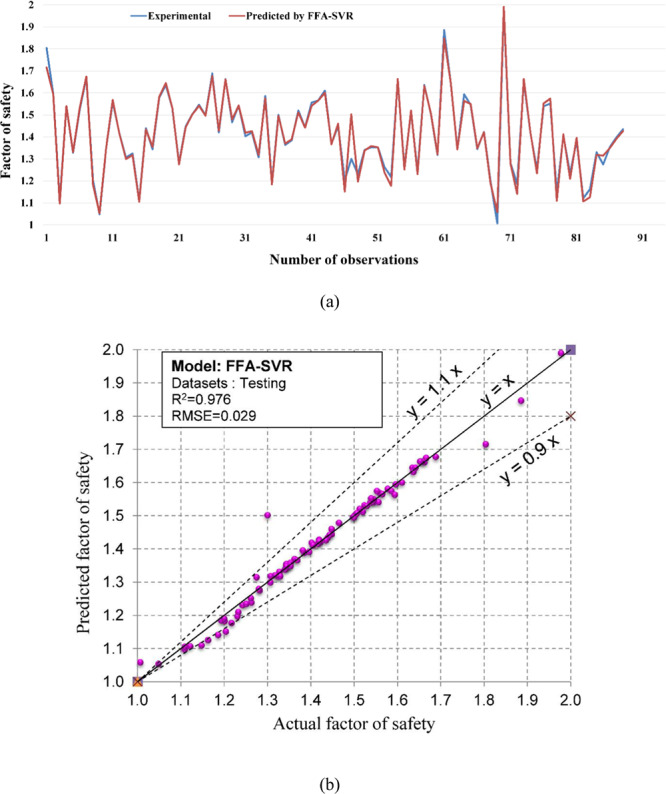
The accuracy and the converging of the FFA-SVR model in predicting FOS. (**a**) Different between the actual and predicted FOS values. (**b**) Correlation analyses of the actual and predicted FOS values.

**Figure 16:**
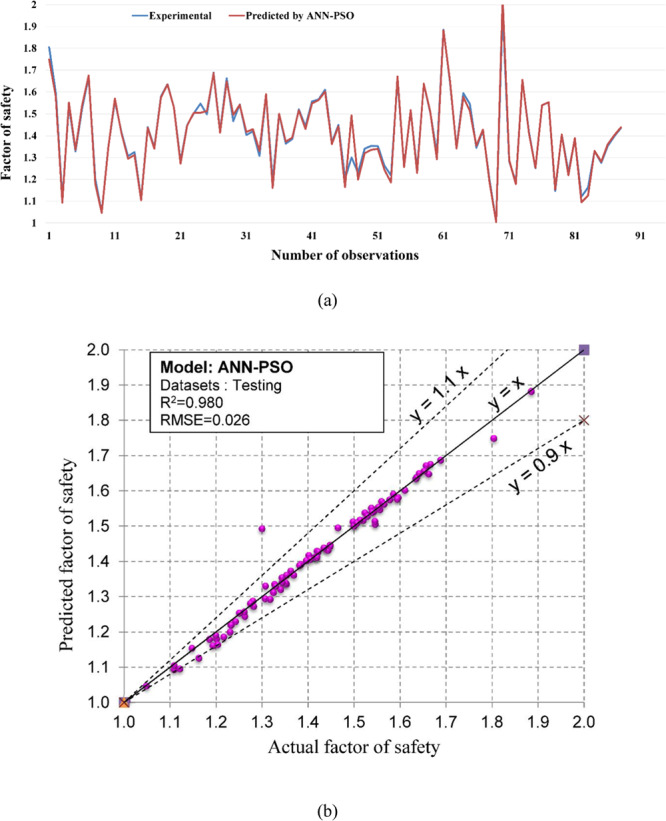
The accuracy and the converging of the ANN-PSO model in predicting FOS. (**a**) Different between the actual and predicted FOS values. (**b**) Correlation analyses of the actual and predicted FOS values.

**Figure 17:**
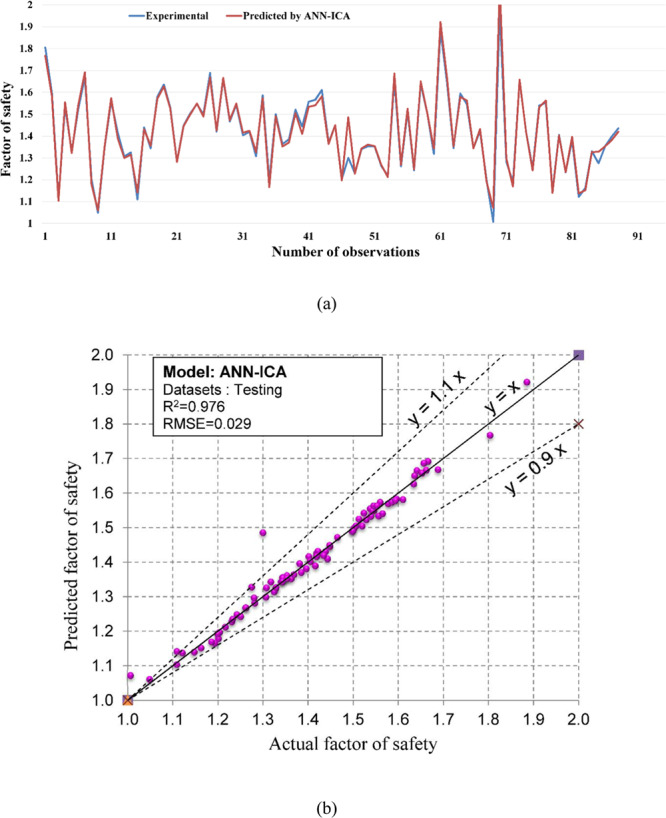
The accuracy and the converging of the ANN-ICA model in predicting FOS. (**a**) Different between the actual and predicted FOS values. (**b**) Correlation analyses of the actual and predicted FOS values.

**Figure 18:**
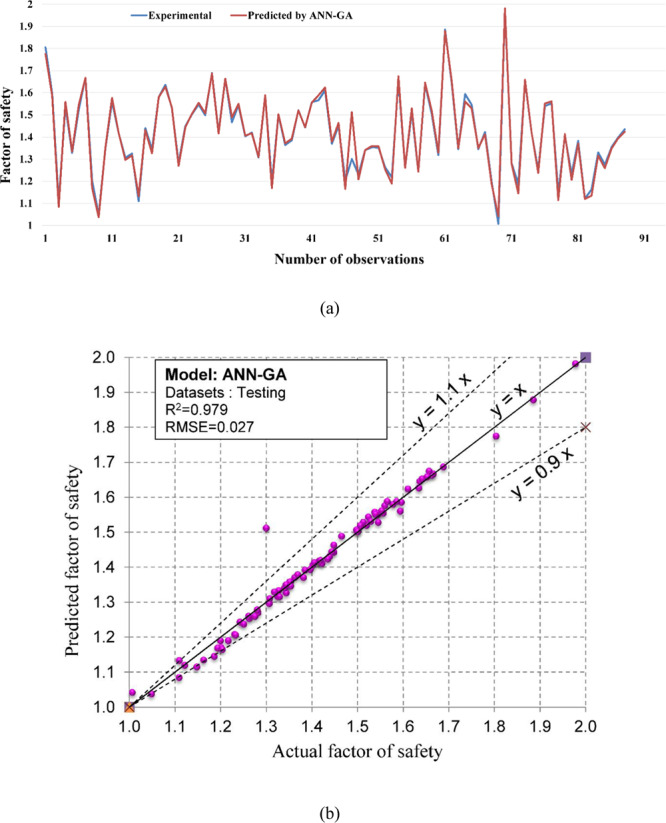
The accuracy and the converging of the ANN-GA model in predicting FOS. (**a**) Different between the actual and predicted FOS values. (**b**) Correlation analyses of the actual and predicted FOS values.

**Figure 19:**
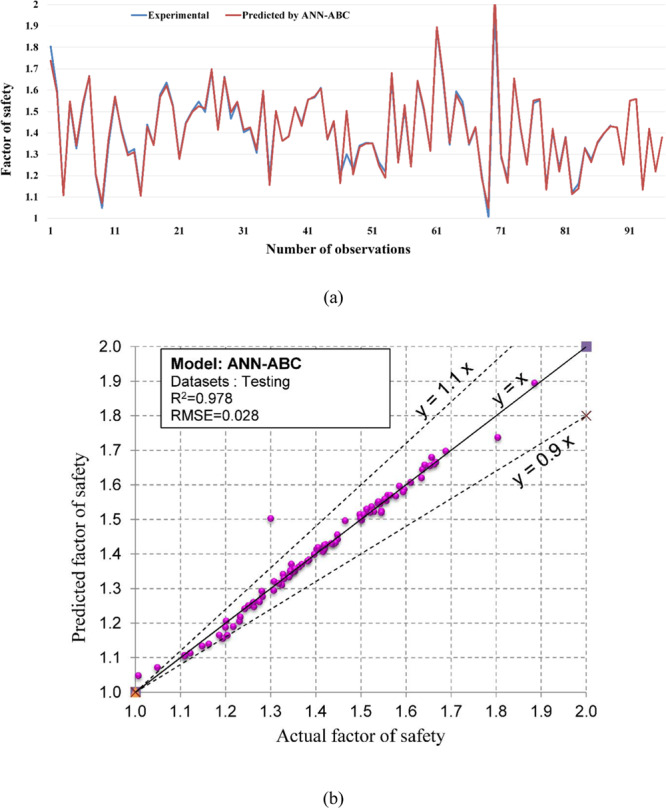
The accuracy and the converging of the ANN-ABC model in predicting FOS. (**a**) Different between the actual and predicted FOS values. (**b**) Correlation analyses of the actual and predicted FOS values.

Regarding the accuracy of the models, a color range can preliminarily provide a performance evaluation of the models. Green and white represent the best and worst performances, respectively. Table 3 showed that the proposed M5Rules–GA model provided the best performance among the developed models in this study. In contrast, the ANN 5-12-16-1 model yielded the worst performance. Considering the accuracy/performance of the proposed M5Rules-GA and previously introduced models (i.e., FFA-SVR, ANN-PSO, ANN-ICA, ANN-GA, and ANN-ABC), it can be seen that the accuracy of the M5Rules-GA model is also higher than the other hybrid models. Indeed, the total ranking of the M5Rules-GA model was 33, whereas the best hybrid model among the FFA-SVR, ANN-PSO, ANN-ICA, ANN-GA, and ANN-ABC models only received a total ranking of 30 (ANN-PSO). It is worth mentioning that the role of the GA is the optimization of parameters of the models (such as M5Rules and ANN). However, we can see that the performance of the M5Rules-GA model is better than the ANN-GA model. This finding indicates that the M5Rules model is fitter than the ANN models. The FOS predictions, correlation schemes, and their 90% confidence level of the models are illustrated in Figs. 9–19.

**Figure 20:**
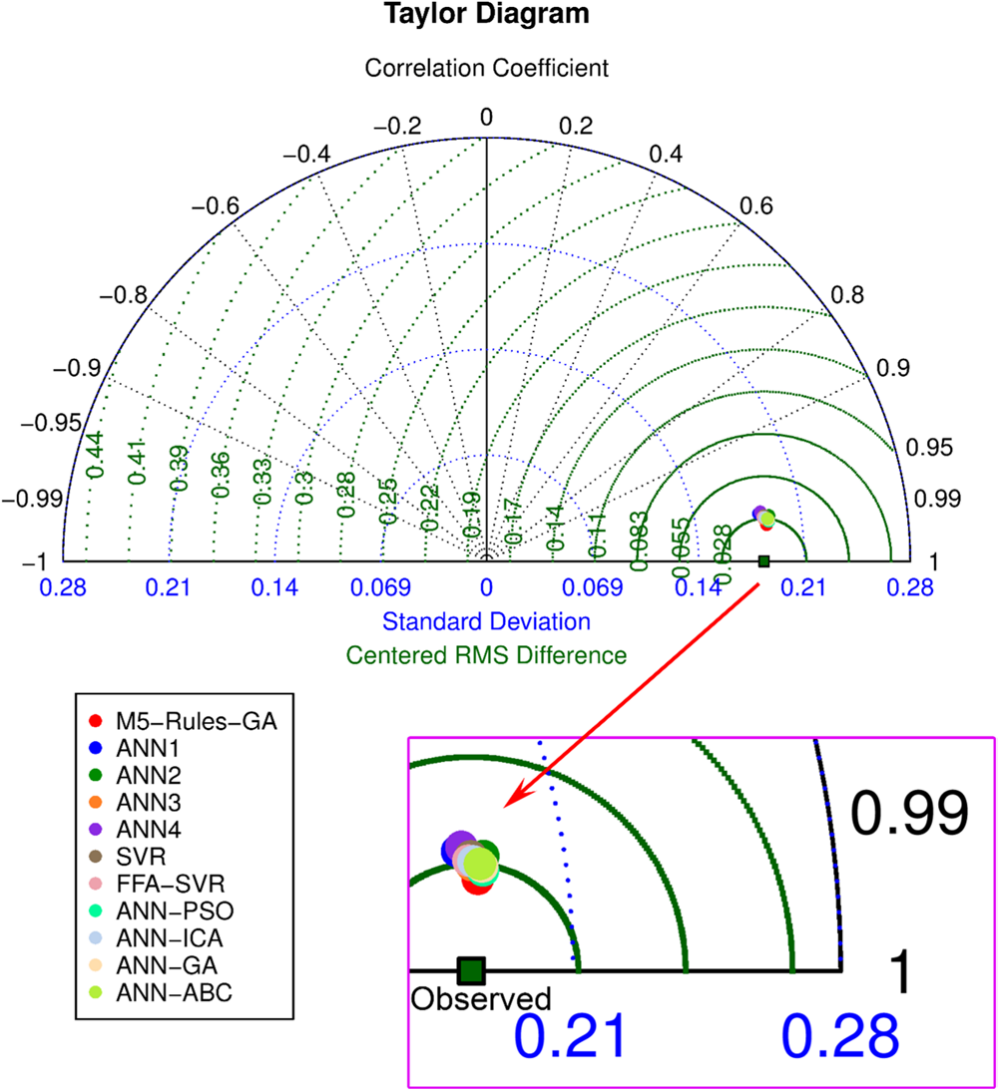
Assessment of the models using Taylor diagram.

Considering further evaluation criteria, such as standard deviation, centered root mean square (RMS) difference, and correlation coefficient, a Taylor diagram was drawn to visualize and comprehensively assess the developed models, as shown in Fig. 20. As seen in the figure, the proposed M5Rules-GA was on the smallest arcs of standard deviation, centered RMS difference, and correlation coefficient. A closer look at the models shows that the standard deviations of the M5Rules-GA and other models do not differ considerably as they seem to be on the same arc. However, centered RMS difference and correlation coefficient of the proposed M5Rules-GA model were superior.

## Conclusions

Based on the results of this study, the proposed M5Rules–GA model provided the best accuracy among all the investigated models for slope stability forecasting. The M5Rules model was substantially enhanced using GA optimization, thereby achieving outstanding performance. We expect M5Rules–GA model to be useful in evaluating and predicting slope stability at mines, thus preventing and minimizing slope collapse damage. In addition, the other models also showed positive results, and they might be considered in other instances. Although the performance of the proposed M5Rules–GA model was interpreted in the context of the present study’s dataset, we suggest its broader application to other regions with extended datasets.
